# Pharmacokinetic Monitoring of Methotrexate in Haematological Patients: Comparison of the Predictive Ability of Two Population-Based Models and Their Clinical Utility

**DOI:** 10.3390/pharmaceutics18101255

**Published:** 2026-10-02

**Authors:** María López-Galán, Aida López-Gómez, Maria Antonia Pérez-Moreno, Marta Mejías-Trueba, María Dolores Vega-Coca, Laura Herrera-Hidalgo

**Affiliations:** 1 Department of Pharmacy, Virgen Del Rocío University Hospital, 41013 Seville, Spain; 2 Department of Infectious Diseases, Microbiology and Parasitology, Virgen Del Rocío University Hospital, 41013 Seville, Spain; 3 Institute of Biomedicine of Seville, Virgen Del Rocío University Hospital/CSIC/University of Seville, 41013 Seville, Spain; 4 Centro de Investigación Biomédica en Red de Enfermedades Infecciosas (CIBERINFEC), 28029 Madrid, Spain

**Keywords:** methotrexate, population pharmacokinetics, Bayesian forecasting, folinic acid rescue, lymphoma, acute lymphoblastic leukaemia

## Abstract

**Background and Objectives:** Methotrexate (MTX) is a key drug for treating haematologic malignancies. Due to its narrow therapeutic window, Population pharmacokinetic (PopPK) models help predict MTX plasma concentrations (Cp) and optimize folinic acid rescue. This study evaluated and compared the predictive performance and clinical utility of two PopPK models in adults with lymphoma or acute lymphoblastic leukaemia (ALL). **Methods:** A retrospective PK study was conducted in adults with lymphoma/ALL treated with MTX. Cp at 24 h (lymphoma) and 42 h (ALL) were estimated by Bayesian forecasting using the Gallais et al. (2021) and Kawakatsu et al. (2019) PopPK models with Levenberg–Marquardt least squares (M2) and Strict Adaptive Metropolis (SAA), using frequency-based (FB) or concentration-based (CB) weighting. Predictive performance was assessed using median percentage error (MDiPE), median absolute percentage error (MDiAPE), and the percentage of individual prediction errors within ±20% (iF20) and ±30% (iF30) of observed concentrations. Clinical utility was evaluated by concordance with folinic acid rescue. **Results:** Thirty-seven patients (72 MTX cycles) were included: 17 with lymphoma (37 cycles; 52% men; median age of 66 (34–73) years) and 20 with ALL (35 cycles; 55% men; median age of 46 (17–68) years), with a median of two cycles per patient. In lymphoma, the Kawakatsu model showed the most favourable predictive performance with SAA-FB (MDiPE of 8.5%, MDiAPE of 30.3%, iF20 of 37.83%, and iF30 of 48.65%), whereas the Gallais model showed lower predictive accuracy across the evaluated approaches. Concordance with folinic acid rescue ranged from 75.68% to 86.49%, with the highest value observed with M2-CB. In ALL, both models showed lower and more variable predictive performance, with MDiAPE ranging from 43.0% to 73.8%, iF30 from 11.43% to 42.86%, and concordance with folinic acid rescue from 40.00% to 54.29%. **Conclusions:** PopPK models for MTX showed variable performance in external cohorts, with numerically more favourable results for the model by Kawakatsu et al. External validation is essential prior to clinical application, particularly given the relationship between MTX exposure, delayed clearance and toxicity. Validated models could contribute to safer monitoring and more personalised treatment, although larger studies are needed to confirm their clinical utility.

## 1. Introduction

Methotrexate (MTX) is an antimetabolite widely used in the treatment of haematological malignancies, especially acute lymphoblastic leukaemia (ALL) and lymphomas. It acts by inhibiting dihydrofolate reductase, thereby interfering with nucleic acid synthesis, which hinders the proliferation of malignant haematological cells [1,2,3]. In these cases, high doses of MTX (HD-MTX) are used, defined as those equal to or greater than 500 mg/m^2^ administered intravenously [4]. HD-MTX is associated with significant toxicity—nephrotoxicity, hepatotoxicity, neurotoxicity, mucositis, and myelosuppression—which can lead to treatment delays; prolonged hospitalisations; or, in extreme cases, treatment-related mortality [4,5]. To prevent it, supportive measures are widely implemented. Those measures include intensive intravenous hydration, urinary alkalinisation, folinic acid rescue, and exhaustive MTX plasma concentration (Cp) and renal function monitoring [4].

Despite these strategies, MTX toxicity remains frequent due to substantial interindividual variability in clearance. In adult patients, population-based studies have shown that individuals with higher body surface area (BSA), especially overweight patients, tend to exhibit higher MTX Cp and an increased risk of acute kidney injury, highlighting the limitations of BSA-based dosing strategies in this population [6]. In paediatric populations, in addition to renal function and BSA, haemoglobin levels have been identified as significant predictors of MTX clearance, explaining a relevant proportion of its variability and further illustrating the complexity of MTX disposition [3]. Moreover, population pharmacokinetic (PopPK) models developed in mixed paediatric–adult cohorts consistently identify glomerular filtration rate (eGFR) as the main determinant of MTX clearance, which may vary more than two-fold across the observed range of renal function, underscoring the need for more accurate and individualised predictive approaches [5].

Therapeutic drug monitoring (TDM) is therefore essential in HD-MTX therapy to detect delayed elimination and to guide folinic acid rescue adjustment [4]. However, TDM is inherently reactive, as it relies on measured Cp obtained after drug administration and provides limited ability to anticipate systemic exposure or toxicity risk [1,4]. In this context, model-informed precision dosing (MIPD), which integrates PopPK models with Bayesian forecasting, has emerged as a promising strategy to individualise HD-MTX therapy and optimise supportive care by predicting future concentrations from sparse sampling data [7]. Clinical studies conducted in adult patients receiving HD-MTX have demonstrated that MIPD improves the prediction of delayed MTX clearance and supports earlier and more targeted clinical interventions compared with conventional TDM approaches [1]. Nevertheless, PopPK model performance is population-dependent, and external evaluations have shown that predictive accuracy decreases when models are extrapolated to independent cohorts. However, Bayesian estimation using individual patient data significantly improves predictive performance, underscoring the need for external validation prior to clinical application [8,9].

At present, no standardized TDM-MIPD approach for MTX in patients with haematologic malignancies has been established in routine clinical practice in our setting. In this context, following a review of the literature, two population PopPK models that could be implemented in routine clinical practice for MTX monitoring were identified [5,6]. The characteristics of the study population and treatment regimens in both models closely resemble those of patients treated in our centre, making them appropriate candidates for external validation in our cohort.

Therefore, this study aims to evaluate the predictive capacity of two MTX PopPK models to estimate HD-MTX Cp in adults with haematological malignancies, with a particular focus on their clinical applicability—specifically, assessing whether these models can effectively support individualised therapeutic decision-making to improve patient safety and treatment outcomes.

## 2. Methodology

A retrospective observational study was carried out, including all patients with haematological diseases—specifically, lymphoma or ALL—who received MTX treatment during 2024 at the Hospital Universitario Virgen del Rocío, Seville, Spain.

### 2.1. External Validation Cohort

For model validation, demographic, clinical and laboratory data were collected. Demographic variables included sex, age, weight, and height. Clinical variables comprised diagnosis, number of treatment cycles administered, MTX dose, and infusion duration (4 h in lymphoma and 24 h in ALL). Considered laboratory variables were serum creatinine, alanine aminotransferase (ALT), and plasma methotrexate concentrations measured at the different times established according to the pathology.

#### 2.1.1. Lymphoma Cohort

In patients diagnosed with lymphoma, an institutional treatment protocol was developed based on international guideline recommendations [10,11], including the management of rescue therapy. According to this protocol, MTX was administered at a dose of 2–3 g/m^2^ as a 4 h intravenous infusion.

In this cohort, rescue therapy with folinic acid was initiated 24 h after MTX administration. The dosing regimen was adjusted according to the real drug Cp measured at protocol-defined monitoring time points once the results became available. In patients with normal MTX clearance, the standard dose was 15 mg/m^2^ of folinic acid administered intravenously every 6 h. In situations of severe toxicity or delayed drug elimination, the folinic acid regimen was individually adjusted according to plasma MTX concentrations (Table 1).

MTX Cp were determined at different time points—specifically, 4, 12, 24, 42, and 66 h after the start of infusion. Monitoring was continued with additional measurements every 24 h until plasma concentrations below 0.2 µmol/L were achieved.

In addition, institutional HD-MTX protocols include standardised support measures, such as intensive hydration and systematic monitoring of urinary pH, which are only adjusted or intensified in cases where plasma MTX concentrations predict an increased risk of toxicity. In any case, when adjustment of these supportive measures is required, the protocol specifies that interventions should generally be implemented at 24 or 42 h. Therefore, the concentrations obtained before these time points and used for model prediction are unlikely to be affected. The protocols also list the drugs that are contraindicated during HD-MTX administration.

#### 2.1.2. Lymphoblastic Leukaemia Cohort

In the cohort of patients with acute ALL, treatment was administered according to the protocol [10,12]. The dose of MTX used ranged from 1 to 5 g/m^2^, administered on a 24 h schedule. Initially, an intravenous bolus corresponding to 10% of the total dose was administered during the first 30 min, followed by a continuous infusion of the remaining 90% over a period of 23.5 h.

Unlike the protocol established for the lymphoma cohort, in patients with ALL, folinic acid rescue was initiated 42 h after the start of MTX administration, with a dosage adjustment based on the drug’s Cp determined at that time (Table 1).

MTX Cp monitoring was performed by serial determinations 12, 23, 36, 42, and 60 h after the start of the infusion, with the possibility of extending the measurements at 24 h intervals until Cp below 0.2 µmol/L were reached.

### 2.2. Bayesian Forecasting

MTX Cp were estimated by Bayesian forecasting, considering both the individual and the population characteristics. For this purpose, the performance of two PopPK models was analysed: the model developed by Kawakatsu et al. (2019) [5] and the model proposed by Gallais et al. (2021) [6].

The population pharmacokinetic model developed by Kawakatsu was based on a retrospective cohort of 320 paediatric and adult patients diagnosed with oncological and haematological diseases (including osteosarcoma, lymphoma, and LLA, among others) treated with HD-MTX (≥500 mg/m^2^). This model has a two-compartment structure with linear elimination. The covariates identified as independent predictors of clearance (CL) were estimated eGFR and number of treatment cycles. Similarly, age and alanine aminotransferase (ALT) were associated with central distribution volume (V1), while ALT and the presence of osteosarcoma were related to peripheral distribution volume (V2) [5].

The population pharmacokinetic model proposed by Gallais was developed from a single-centre retrospective study involving 328 adult patients diagnosed with ALL or non-Hodgkin lymphoma (NHL) treated with HD-MTX (≥1 g/m^2^). The population analysis was performed using a two-compartment model with linear elimination. Interindividual variability was incorporated for CL and V2, while intercompartmental clearance (Q) was set at 0.15 L/h due to insufficient data in the initial sampling phase. The model identified age as the main covariate associated with CL and body weight as a determinant of V2 [6]. A summary of the main characteristics and parameters of the two population pharmacokinetic models is provided in Supplementary Table S1.

For the Bayesian forecasting, two optimisation methods were applied with the aim of estimating individual pharmacokinetic parameters, using the structural pharmacokinetic model, simulated population parameters and experimental concentrations as a reference. The evaluated algorithms were the Strict Adaptive Metropolis algorithm (SAA) and the Levenberg–Marquardt-based least squares method (M2). The SAA begins by setting the pharmacokinetic parameters to the average values for the population, then generates iterations from distributions centred on the current parameters, applying the Metropolis-Hastings acceptance or rejection criterion. In its strict version, the variations from the initial population values are minimal. The M2, on the other hand, is a least squares optimisation method that provides the best fit of the pharmacokinetic parameters to the measured concentrations. This procedure uses the mean and variance of the population parameters as limits of the search space, together with the extreme value threshold set at 99%.

The data were weighted using one of two approaches: a frequency-based (FB) or concentration-based (CB) method. The FB method assigns greater weight to more recent concentrations, thereby increasing the accuracy of the estimate, while the CB method gives greater relevance to higher concentrations in order to minimise the overall difference between observed concentrations (Cobs) and forecasts. For CB weighting, the cost function was defined as cost = ∑_i_ (estimated_i_ − measured_i_)^2^, where estimated_i_ and measured_i_ represent the predicted and observed MTX concentrations, respectively. For FB weighting, the cost function was defined as cost = ∑_i_ ((estimated_i_ − measured_i_) · (time_i_ + 1))^2^, where time_i_ represents the sampling time in hours.

Simulations and calculations were performed using Rx Studio pharmacokinetic software 1.57.5.

#### External Evaluation of Predictive Performance

For evaluation of the predictive performance of the selected models, plasma MTX levels at 4 and 12 h (C4 h and C12 h) in lymphoma patients and concentrations at 12, 23 and 36 h (C12 h, C23 h and C36 h) in ALL patients were used to predict concentrations at predetermined times. Concentrations affected by confirmed or suspected errors in sample collection or processing, including samples likely collected from the MTX infusion line during the 24 h infusion in ALL patients, were excluded. These exclusions were performed before evaluation of model predictive performance.

Each treatment cycle was treated as an independent observation for the calculation of the predictive performance and agreement measures.

The individual predicted concentration (iCpred) was compared with the observed concentrations (Cobs) at the time points prior to the start of folinic acid rescue therapy, set at 24 h for patients with lymphoma and 42 h for those with ALL.

To assess the accuracy of the pharmacokinetic models, the individual percentage prediction error (iPE) was calculated for each patient using the following formula [11,13]:

iPE (%) = (iCpred − Cobs) × 100  Cobs

From the iPE values obtained for the entire cohort, the median individual percentage prediction error (MDiPE) was calculated and used as a measure of overall model accuracy. A median error ≤ 20% was considered as a target value, accepting a maximum error of 30% [12,13].

Model accuracy was assessed by the median absolute percentage error of prediction (MDiAPE), calculated from the individual absolute percentage error (iAPE) for each patient, according to the following formula [11,13]:

AiPE (%) = I iCpred − Cobs I × 100  Cobs

The target value for the MDiAPE was a median error ≤ 30%, with an upper limit of 35% error [11,13].

Additionally, the percentage of predictions with an error less than or equal to 20% (iF20) and 30% (iF30) was calculated. These indicators represent the fraction of iPE whose value is in the ranges of 80–120% (iF20) and 70–130% (iF30) with respect to the Cobs. The reference values considered acceptable were: >30% of predictions within the defined range for iF20 and >45% for iF30 [11,13].

In order to assess the clinical utility of both models in adjusting the folinic acid rescue dose defined in the protocol, the agreement between the recommended dose from the iCpred and the corresponding dose from the Cobs was analysed (recommended dose higher, lower than or equal to the actual dose).

### 2.3. Statistical Analysis

Descriptive statistics were used for categorical variables, which were summarised as frequency measures. For continuous quantitative variables, distribution assumptions were assessed. Data with a normal distribution are expressed as mean ± standard deviation, while data with a non-normal distribution are described as median and interquartile range (IQR). The Shapiro–Wilk test was used to assess normality. Data were analysed using IBM SPSS Statistics, version 25.

## 3. Results

### 3.1. Demographic and Clinical Characteristics of the Study Population

This study included a total of 72 cycles of HD-MTX, corresponding to 37 adult patients. In the overall cohort, sex distribution was balanced, with males accounting for 51.4% of the population. Age at diagnosis showed wide variability, with a median of 58 years (IQR 33–68). Most patients received one or two chemotherapy cycles during the study period.

The baseline demographic and clinical characteristics stratified by diagnosis are presented in Table 2. Of the total number of patients included, 17 (45.9%) belonged to the lymphoma cohort and received 37 cycles of treatment, while 20 patients (54.1%) belonged to the acute ALL cohort, with a total of 35 cycles. The distribution by sex was similar in both groups, with a slight predominance of males in the ALL cohort and females in the lymphoma cohort. ALL patients were generally younger and had a wider age range than lymphoma patients. Anthropometric variables showed comparable values between the two cohorts, although lymphoma patients tended to have slightly higher values. In terms of baseline renal function, patients with ALL had lower serum creatinine concentrations. With regard to treatment, MTX doses adjusted for body surface area were higher in the lymphoma cohort, while in the ALL cohort, there was greater variability in the administered doses.

### 3.2. Model Performance and Clinical Application

Table 3 and Table 4 summarise, for each model–method combination (SAA and M2; FB and CB), the corresponding predictive accuracy metrics and their clinical implications, evaluated in terms of the ability to adequately estimate the need for folinic acid rescue and the concordance between the predicted and the actual folinic acid doses administered in both study populations (24 h and 42 h MTX Cp after 4 h and 24 h HD-MTX infusions in lymphoma and ALL patients, respectively).

**Figure 1:**
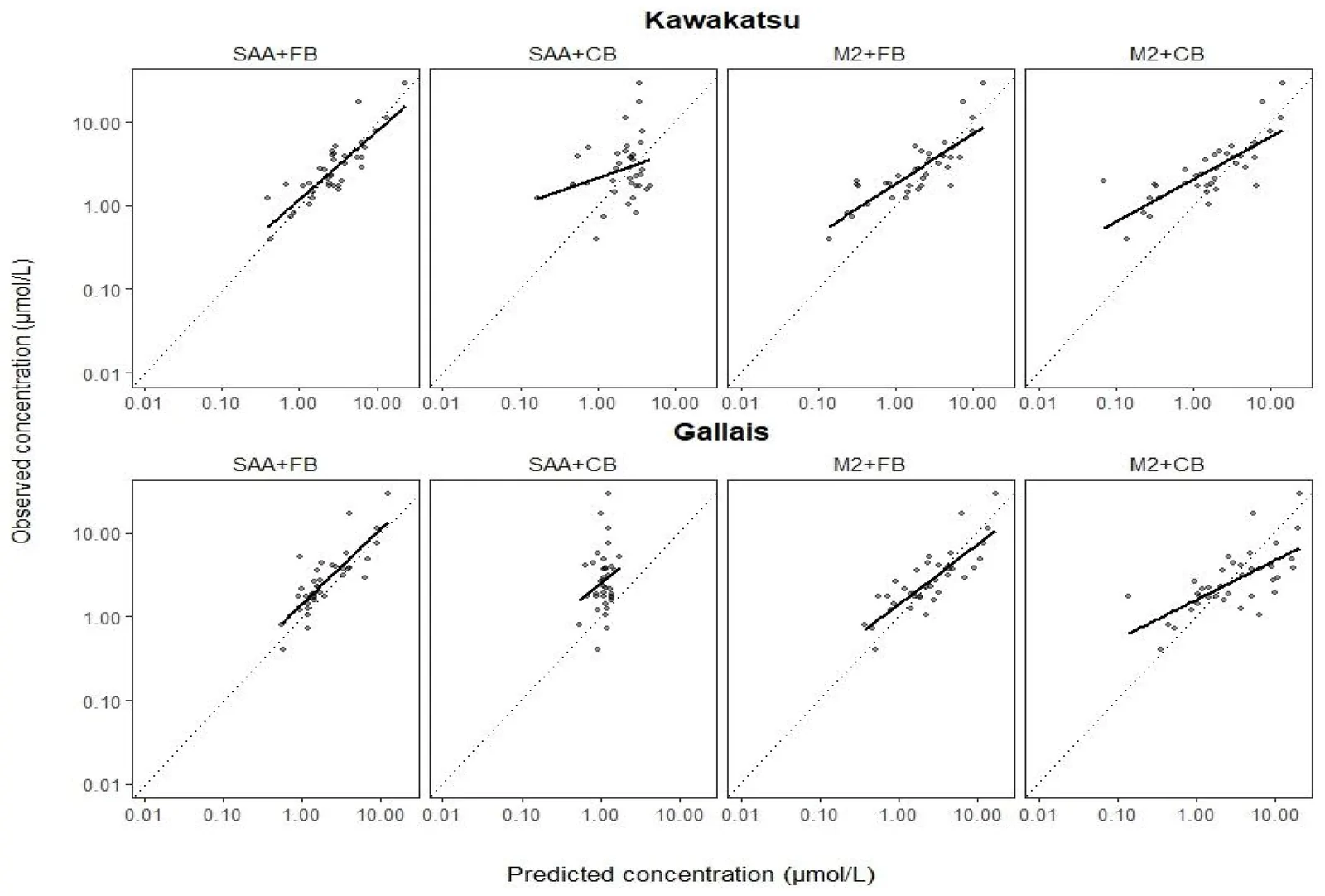
Observed versus predicted MTX concentrations at 24 h. SAA, Strict Adaptive Metropolis algorithm; M2, Levenberg–Marquardt-based least squares method; CB, concentration-based; FB, frequency-based.

**Figure 2:**
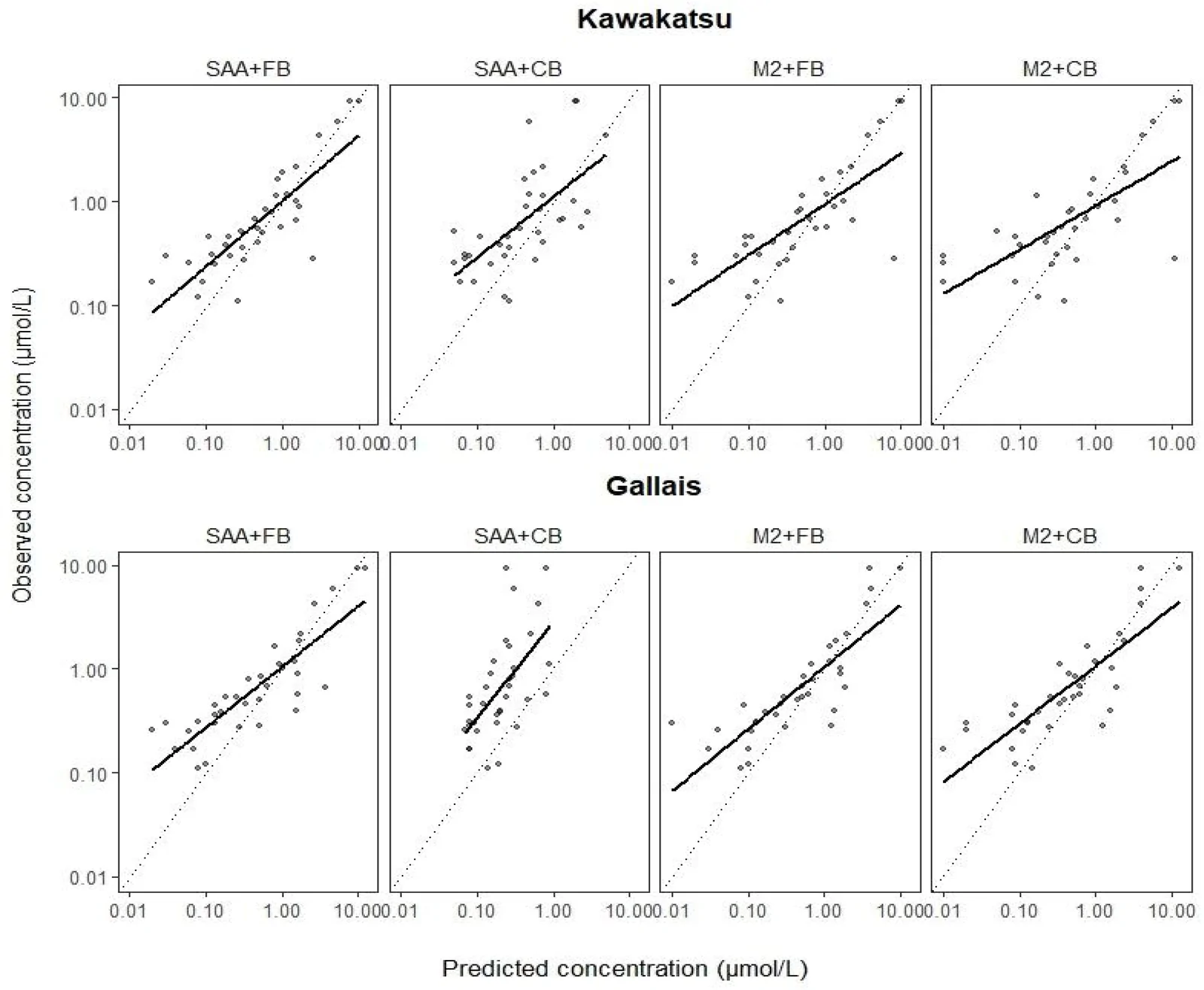
Observed versus predicted MTX concentrations at 42 h. SAA, Strict Adaptive Metropolis algorithm; M2, Levenberg–Marquardt-based least squares method; CB, concentration-based; FB, frequency-based.

The agreement between observed and model-predicted MTX Cp values is further illustrated by the observed-versus-predicted concentration plots shown in Figure 1 and Figure 2.

#### 3.2.1. Predictive Performance at 24 and 42 h

Predictive performance of the Kawakatsu and Gallais models varied according to the optimisation algorithm, weighting strategy, and sampling time (Table 3 and Table 4).

At 24 h in patients with lymphoma receiving short HD-MTX infusions, the Kawakatsu model combined with SAA optimisation and FB weighting showed a prediction bias of 8.5% (MDiPE), a precision of 30.3% (MDiAPE) and the highest iF20 (37.8%) and iF30 (48.7%), fulfilling all predefined accuracy criteria. In contrast, application of the same configuration to the Gallais model was associated with underprediction (MDiPE −21.7%) and lower accuracy (iF20 32.4%). Strategies combining SAA with CB weighting yielded marked performance deterioration for both models at this time point, particularly for the Gallais model (MDiPE −56.8%, iF20 ≤ 13.5%). Approaches based on the M2 algorithm showed intermediate performance at 24 h; the Kawakatsu model with FB weighting achieved an MDiPE of −23.3% and an iF20 of 27.0%, while CB weighting reduced bias but did not surpass the accuracy of the SAA + FB–Kawakatsu configuration.

At 42 h in patients with ALL receiving prolonged HD-MTX infusions, overall predictive accuracy was lower and more heterogeneous than at 24 h, and none of the model–method combinations met all predefined accuracy criteria. Configurations based on M2 generally showed comparatively better performance; the Kawakatsu model with FB weighting provided the lowest prediction bias (MDiPE −13.3%) and the highest iF20 (34.3%), although precision was limited (MDiAPE 43.0%) and iF30 (37.1%) remained below the predefined threshold. Under the same configuration, the Gallais model showed greater underprediction (MDiPE −27.3%) and lower accuracy (iF20 28.6%). SAA-based strategies, particularly SAA + CB, showed the poorest performance at 42 h, with the Gallais model presenting the largest deviations (MDiPE of −69.0%, MDiAPE of 69.0%, iF20 ≤ 5.7%, and iF30 of 11.4%). With M2 + CB, prediction bias for the Kawakatsu model was close to the target range (MDiPE of −4.6%), but precision and accuracy remained below the predefined thresholds.

Observed-versus-predicted concentration plots at 24 and 42 h (Figure 1 and Figure 2) were consistent with these quantitative metrics. Configurations with lower prediction bias and higher accuracy indices showed closer clustering of points around the line of identity, whereas those with higher prediction error exhibited increased dispersion and systematic deviation from the diagonal.

#### 3.2.2. Folinic Acid Rescue

Regarding folinic acid rescue, concordance between model-based predictions and administered doses was higher for the Kawakatsu model than for the Gallais model at both time points. At 24 h, the highest concordance was observed with the Kawakatsu model under M2 + CB (86.49%), with other Kawakatsu-based configurations (SAA + FB, SAA + CB, and M2 + FB) also showing high agreement (75.68–83.78%). Gallais-based strategies showed concordance values between 64.86% and 81.08%. At 42 h, concordance ranged from 37.14% to 54.29%, with the highest values consistently observed for the Kawakatsu model combined with M2 + FB, M2 + CB or SAA + FB (all 54.29%), whereas the Gallais model showed lower concordance (37.14–51.43%), with its best performance under M2 + FB.

Clinically relevant dosing errors were generally infrequent but showed different patterns across strategies and time points. Overestimation of folinic acid rescue doses occurred in ≤11% of cases at 24 h and in ≤20% at 42 h and was not observed for the SAA + CB configuration at 24 h or for the Gallais model with SAA + CB at 42 h. The highest overestimation rate was recorded for the Kawakatsu model with M2 + CB at 42 h (20%). Underestimation occurred less frequently but was more variable: at 24 h, the lowest underestimation rates (5.41%) were observed for the Kawakatsu model with M2 + CB and SAA + FB, whereas higher and more consistent underestimation was observed for the Gallais model across strategies. At 42 h, underestimation rates ranged from 25.71% to 62.86%, with the lowest values for the Kawakatsu model combined with M2 + CB (25.71%), M2 + FB (28.57%) and SAA + FB (31.43%) and the highest for the Gallais model with SAA + CB (62.86%).

## 4. Discussion

HD-MTX administration presents high pharmacokinetic variability and a narrow therapeutic margin [7], requiring TDM to optimize its safety and efficacy [9]. In this context, PopPK models, together with Bayesian forecasting, allow for the prediction of individual MTX elimination and support clinical decisions, including the adjustment of folinic acid rescue therapy [14]. However, as there is currently no reference PopPK model for adult patients with ALL or lymphoma and because model utility depends on the generation of reliable predictions in external populations, external validation is essential before clinical implementation to avoid inappropriate therapeutic decisions [14,15,16]. In this regard, the present study externally evaluated two PopPK models in an adult cohort of patients with ALL and lymphoma treated with HD-MTX, analysing their ability to predict plasma MTX concentrations before initiation of folinic acid rescue and their potential clinical impact.

The results of external validation revealed substantial variability in predictive performance across diagnostic cohorts and optimisation strategies. In both lymphoma and ALL populations, the model developed by Kawakatsu et al. showed greater predictive accuracy than the model developed by Gallais et al. Similarly, estimates were generally more accurate when FB weighting was applied rather than CB weighting. Differences were also observed depending on the pathology: in lymphoma patients treated with short MTX infusions, the SAA algorithm showed the best performance, especially when combined with the Kawakatsu et al. model and FB weighting, showing low bias and a higher proportion of predictions within the predefined error margins. In contrast, among patients with ALL receiving prolonged 24 h infusions, the M2 method offered relatively more favourable results. Overall, both models performed more favourably in the lymphoma cohort, whereas predictive accuracy was lower and more heterogeneous in the ALL cohort, with a general tendency to underestimate plasma MTX concentrations.

These findings are consistent with previous studies. For example, Chen et al. [15] found that none of the seven evaluated PopPK models met predefined external validation criteria. In that study, the model developed by Gallais et al. showed high bias and low accuracy, limiting its clinical applicability. In this context, the model developed by Kawakatsu et al. has demonstrated better performance in external validations studies, especially during the elimination phase of MTX disposition [16].

Although both models were developed for populations similar to that evaluated in the present study, some differences among them and relative to our cohort could explain the observed predictive performance. Both models employ a two-compartment structure, which has been previously considered adequately to characterize MTX pharmacokinetics [8]. However, important differences exist regarding the covariates included to describe MTX clearance. The Kawakatsu model incorporates eGFR, a covariate considered more informative than serum creatinine alone [5], as well as the number of MTX treatment cycles. In contrast, the Gallais model identifies age as the only covariate influencing clearance, which might be insufficient to capture the complexity of MTX elimination [17,18,19]. In our cohort, the median number of administered MTX cycles was 1 in patients with ALL and 2.41 in those with lymphoma, considerably lower than the median of 4 reported by Kawakatsu et al. The effect of repeated MTX cycles has been previously described, suggesting a decrease in clearance with repeated exposure to the drug [17]. Therefore, differences in treatment exposure may have contributed to the reduced predictive accuracy observed in our population.

The models also differ in terms of the population and sampling strategies used for their development. Kawakatsu et al. developed their model using a multicentre and heterogeneous cohort that included adults and children with ALL, lymphoma, and osteosarcoma [5], while Gallais et al. used a single-centre cohort of adults with ALL and NHL [6], which could limit its generalisation to external populations [7,14,15]. Furthermore, Kawakatsu’s model was developed with a limited number of initial time points, mainly in the paediatric population, and only 5.5% of concentrations were obtained during MTX infusion, which may have contributed to the underestimation of initial MTX concentrations [5]. Similarly, the model by Gallais et al. did not include any measurements during infusion [6]. In our study, predictions at 42 h in the ALL cohort included two samples obtained during infusion. Taken together, these differences in population characteristics, covariates, and sampling design could explain the observed underestimation and the lower predictive performance of both models in our cohort.

From a clinical perspective, this study not only evaluated the statistical accuracy of the predictions but also their potential impact on therapeutic decision-making related to folinic acid rescue by comparing the rescue requirement estimated from predicted versus observed MTX concentrations. This approach allowed us to determine whether model-based predictions would result in a higher, lower, or equivalent folinic acid rescue requirement, an aspect with direct clinical implications that has hardly been explored in previous external validation studies. Consequently, opportunities for comparison with published evidence remain limited. In HD-MTX regimens administered as a 4 h infusion, most predictions led to adequate adjustments of folinic acid rescue for both models, except for the CB approach using the M2 optimisation method in the Gallais et al. model, where this ratio was reduced, in association with a significant overestimation of rescue. In contrast, in the cohort of patients treated with MTX in a 24 h infusion, the proportion of adequate rescue estimates was significantly lower in both models, with a predominance of underestimation of the need for folinic acid rescue, which is particularly relevant from a clinical point of view, given that an insufficient dose of folinic acid can significantly increase the risk of severe MTX-associated toxicity [9,16]. These results underscore that a good statistical fit of the model does not always translate into a safe therapeutic decision, especially in subjects with delayed elimination, and underscore the need to include, in the external validation of PopPK models, their impact on key therapeutic decisions before their implementation in therapeutic monitoring protocols.

Several limitations should be considered when interpreting the results of this study. First, it is a retrospective design based on electronic clinical records, which may introduce uncertainty and potential information bias. In order to minimise this risk, all data were independently reviewed by two researchers. Second, this was a single-centre study with a limited sample size and some heterogeneity in the available data. Furthermore, the different treatment cycles for the same patient were analysed as independent observations; consequently, any potential intra-patient correlation was not taken into account. Given the limited sample size, it was not possible to carry out analyses stratified by cycle number or at the patient level. Furthermore, as the pharmacokinetics of MTX may vary between successive cycles [17], the possible influence of repeated cycles on the model’s performance cannot be ruled out. However, the real-world nature of the cohort provides clinically relevant information regarding model performance in routine practice. Additionally, the performance thresholds for prediction were based on previously published criteria that are not specific to MTX, as there are no universally accepted standards for this drug. Furthermore, these thresholds are symmetrical and therefore do not account for the potentially different clinical consequences of overestimating or underestimating MTX exposure, particularly given the potential clinical implications of underestimating exposure for folinic acid rescue. Lastly, no pharmacogenetic information was available for the included patients. Therefore, although pharmacogenetic variability could potentially contribute to interindividual differences in MTX pharmacokinetics, its contribution to the differences observed in model performance cannot be established. Currently, the study of polymorphisms in transmembrane transporters (e.g., ATP-binding cassette subfamily B member 1 [ABCB1], ATP-binding cassette subfamily G member 2 [ABCG2]) or enzymes involved in the pharmacokinetics of MTX (e.g., methylenetetrahydrofolate reductase [MTHFR]) is not included in routine clinical practice within our healthcare system’s portfolio of services.

## 5. Conclusions

In conclusion, this study demonstrates that the performance of PopPK models of MTX can vary significantly when applied to external populations, even in clinical settings similar to those in which they were originally developed. Although the model proposed by Kawakatsu et al. showed numerically more favourable overall performance than that developed by Gallais et al. in the evaluated populations, this finding should be interpreted with caution, given the substantial differences between the lymphoma and ALL cohorts and the relatively limited sample size. From a clinical perspective, these results highlight the need to externally validate PopPK models before they are incorporated into routine clinical practice. This consideration is particularly relevant in the case of MTX, given that high exposure and delayed elimination are associated with an increased risk of toxicity. In this context, inaccurate predictions could lead to an inappropriate estimation of drug exposure and influence decisions regarding dose adjustment, rescue measures and clinical monitoring. Conversely, properly validated models could optimise pharmacokinetic monitoring, facilitate the early identification of patients at risk of delayed clearance and toxicity, and contribute to a safer and more individualised treatment strategy. Further studies in larger populations are needed to confirm their clinical utility.

## Figures and Tables

**Table 1:** MTX levels at 24 h with corresponding folinic acid dose adjustment in 4 h MTX cycle and MTX levels at 48 h with corresponding folinic acid dose adjustment in 24 h MTX cycle [10,11].

Folinic Acid Dosage	[MTX] 24 h	[MTX] 48 h
15 mg/m^2^/6 h IV	<5 µM	0.2–1 µM
30 mg/m^2^/6 h IV	5–10 µM	1–2 µM
45 mg/m^2^/6 h IV	**-**	2–3 µM
60 mg/m^2^/6 h IV	**-**	3–4 µM
75 mg/m^2^/6 h IV	**-**	4–5 µM
100 mg/m^2^/3 h IV	10–20 µM	5–10 µM
200 mg/m^2^/6 h IV	**-**	10–20 µM
250 mg/m^2^/6 h IV	20–30 µM	**-**
500 mg/m^2^/6 h IV	30–50 µM	20–50 µM
500 mg/m^2^/3 h IV	50–200 µM	50–100 µM
Assess glucarpidase	>200 µM	>100 µM

MTX, methotrexate.

**Table 2:** Patient demographics and baseline clinical characteristics.

Variable	All Patients (*n* = 37)	ALL (*n* = 20)	Lymphoma (*n* = 17)
Sex (%) Male	19 (51.4%)	11 (55%)	8 (47.0%)
Age at diagnosis (years)	58 (33–68)	46 (25–56)	66 (59–71)
Number of chemotherapy cycles	1.0 (1.0–2.0)	1.0 (1.0–2.0)	2 (1.0–2.5)
Weight (kg)	72.86 ± 17.04	71.86 ± 18.02	74.05 ± 16.28
Height (cm)	166.14± 8.81	166.50 ± 8.29	165.00 ± 9.40
Body surface area (m^2^)	1.80± 0.20	1.78 ± 0.22	1.85 ± 0.19
Serum creatinine (mg/dL)	0.73± 0.20	0.63 (0.51–0.81)	0.82 (0.64–0.93)
Administered dose (mg/m^2^)	2277 (1531–3004)	1522 (1470–3120)	2310 (2190–3002)

ALL, Acute lymphoblastic leukaemia. Note: Age (all patients), weight, height, and body surface area are expressed as mean ± standard deviation. Age, serum creatinine, chemotherapy cycles, and MTX dose are expressed as medians (interquartile range). Sex is reported as a number (percentage %).

**Table 3:** Predictive performance and clinical applicability of MTX concentration estimates at 24 h in patients with lymphoma.

	SAA + FB		SAA + CB		M2 + FB		M2 + CB	
Evaluated model	Kawakatsu	Gallais	Kawakatsu	Gallais	Kawakatsu	Gallais	Kawakatsu	Gallais
Predictive Performance Metrics								
MDiPE (%)	8.50	−21.70	−24.50	−56.80	−23.30	−9.00	−33.30	−10.00
MDiAPE (%)	30.30	32.00	57.10	59.80	31.80	35.60	50.20	42.50
iF20 (%)	37.83	32.43	10.81	13.51	27.02	39.73	21.60	16.22
iF30 (%)	48.65	48.65	21.60	8.11	40.54	37.84	32.43	29.73
Clinical application								
Estimated rescue dose	N (%)							
Overestimated	4 (10.81%)	2 (5.41%)	0 (0%)	0 (0%)	4 (10.81%)	4 (10.81%)	3 (8.11%)	10 (27.03%)
Correct estimation	31 (83.78%)	30 (81.08%)	31 (83.78%)	31 (83.78%)	28 (75.68%)	30 (81.08%)	32 (86.49%)	24 (64.86%)
Underestimation	2 (5.41%)	5 (13.51%)	6 (16.22%)	6 (16.22%)	5 (13.51%)	3 (8.11%)	2 (5.41%)	3 (8.11%)

CB, concentration-based; FB, frequency-based; iF20/iF30, percentage of predictions with an error less than or equal to 20% or 30%; MDiAPE, median absolute percentage error of prediction; MDiPE, median individual percentage prediction error; SAA, Strict Adaptive Metropolis algorithm; M2, Levenberg–Marquardt-based least squares method.

**Table 4:** Predictive performance and clinical applicability of MTX concentration estimates at 42 h in patients with ALL.

	SAA + FB		SAA + CB		M2 + FB		M2 + CB	
Evaluated model	Kawakatsu	Gallais	Kawakatsu	Gallais	Kawakatsu	Gallais	Kawakatsu	Gallais
Predictive Performance Metrics								
MDiPE (%)	−27.30	−26.10	−45.70	−69.00	−13.30	−27.30	−4.60	−26.10
MDiAPE (%)	44.20	53.70	73.80	69.00	43.00	40.20	44.30	50.00
iF20 (%)	25.71	25.71	2.86	5.71	34.29	28.57	28.57	22.86
iF30 (%)	42.86	37.14	11.43	11.43	37.14	42.86	34.29	34.29
Clinical application								
Estimated rescue dose	N (%)							
Overestimated	5 (14.29%)	5 (14.29%)	6 (17.14%)	0 (0%)	6 (17.14%)	5 (14.29%)	7 (20%)	6 (17.14%)
Correct estimation	19 (54.29%)	16 (45.71%)	14 (40%)	13 (37.14%)	19 (54.29%)	18 (51.43%)	19 (54.29%)	16 (45.71%)
Underestimation	11 (31.43%)	14 (40%)	15 (42.86%)	22 (62.86%)	10 (28.57%)	12 (34.29%)	9 (25.71%)	13 (37.14%)

CB, concentration-based; FB, frequency-based; iF20/iF30, percentage of predictions with an error less than or equal to 20% or 30%; MDiAPE, median absolute percentage error of prediction; MDiPE, median individual percentage prediction error; SAA, Strict Adaptive Metropolis algorithm; M2, Levenberg–Marquardt-based least squares method.

## Data Availability

The datasets used and/or analysed during this study are not publicly available due to privacy restrictions.

## Supplementary Materials

The following supporting information can be downloaded at: https://www.mdpi.com/article/10.3390/pharmaceutics18101255/s1, Table S1. Main characteristics of the population pharmacokinetic models evaluated in this study.
