# Supplementary material for: Pharmacokinetic Monitoring of Methotrexate in Haematological Patients: Comparison of the Predictive Ability of Two Population-Based Models and Their Clinical Utility
Source: Pharmaceutics. 2026 Oct 2;18(10):1255. doi: 10.3390/pharmaceutics18101255 (PMC13708096; doi:10.3390/pharmaceutics18101255)
Supplement: Supplementary file 1 [file pharmaceutics-18-01255-s001.zip › pharmaceutics-4572895-supplementary.pdf]

Table S1. Main characteristics of the population pharmacokinetic models evaluated in this study.

**Reference**	**Gallais et al.**	**Kawakastus et al.**
**Country**	France	USA
**Population**	Adults (n=313):<br>ALL: 82<br>Lymphoma: 231	Total n=320<br>Children (n=185) + Adults (n=135):<br>Of the adults<br>- 49 with leukaemia<br>- 75 with lymphoma<br>- 11 with osteosarcoma
**Dose (g/m²)**	1–8 (mean = 3)	1 (ALL), 3 (lymphoma),<br>12 (osteosarcoma)
**Admission time**	22–36 h for ALL<br>0.5–4 h for lymphoma	24 h for ALL<br>3 h for lymphoma<br>4 h for osteosarcoma
**Significant covariate**	CL: age<br>Vp: weight	CL: weight, CLCr, cycles<br>Vc: weight, age, ALT<br>Q: weight<br>Vp: weight, ALT, disease
**CL (L/h)**	8.3	12
**Vc (L)**	27.4	52.1
**Q (L/h)**	0.15	0.13
**Residual variance (%)**	34	43.4
**Model**	Two-compartment structure (linear elimination)	Two-compartment structure
**Plasma concentration**	36 and 48 h, and every 24 h until <0.21	N/A

ALL, acute lymphoblastic leukaemia; CL, clearance; N/A, not available; Q, intercompartmental CL; Vc, central volume of distribution; Vp, peripheral volume of distribution.
